# Pilot Study Regarding Translation of Patient-Oriented Eczema Measure Questionnaire in Romanian Language in Assessing the Correlation Between Quality of Life and Disease Severity in Children with Atopic Dermatitis

**DOI:** 10.3390/children13070905

**Published:** 2026-07-08

**Authors:** Raluca-Gabriela Miulescu, Ioana Roșca, Alexandru-Neculai Pavel, Ruxandra-Cristina Marin, Andreea Teodora Constantin, Florica Sandru, Elena Poenaru, Daniela Eugenia Popescu, Oana Andreia Coman

**Affiliations:** 1 Faculty of Medicine, University of Medicine and Pharmacy “Carol Davila”, 020021 Bucharest, Romania; miulescuraluca@yahoo.com (R.-G.M.); ioana.rosca@umfcd.ro (I.R.); ruxandra.marin@umfcd.ro (R.-C.M.); andreea.constantin@drd.umfcd.ro (A.T.C.); florica.sandru@umfcd.ro (F.S.); oana.coman@umfcd.ro (O.A.C.); 2 Dermatology Department, Saint Constantin Hospital, 500388 Brasov, Romania; 3 Clinical Hospital of Obstetrics and Gynecology “Prof. Dr. P.Sirbu”, 060251 Bucharest, Romania; 4 Pax Clinic, 020951 Bucharest, Romania; alexnpavel@yahoo.com; 5 FutureMeds, 800001 Galati, Romania; 6 Pediatrics Department, National Institute for Mother and Child Health “Alessandrescu-Rusescu”, 20382 Bucharest, Romania; 7 Dermatology Department, Elias University Hospital, 011461 Bucharest, Romania; 8 Department of Obstetrics-Gynecology and Neonatology, “Victor Babeș” University of Medicine and Pharmacy, 300041 Timișoara, Romania

**Keywords:** preterm infants and term infants, atopic dermatitis, SCORAD, POEM

## Abstract

Background: Atopic dermatitis (AD) is a chronic inflammatory skin disease that commonly begins in early childhood and substantially impairs quality of life. Patient-Oriented Eczema Measure (POEM) is a validated patient-reported outcome, whereas SCORing Atopic Dermatitis (SCORAD) is a widely used clinician-assessed severity index for AD. Methods: In this single-center observational analytical pilot study, 90 pediatric patients (1 month–15 years) diagnosed with AD, according to Hanifin and Rajka criteria, were recruited at Saint Constantin Hospital, Brașov, Romania (September 2025–February 2026). During one visit, caregivers and/or children completed the Romanian version of the POEM, and a dermatologist assessed disease severity using SCORAD. Demographic and clinical data were recorded. Descriptive statistics, group comparisons, linear regression, and correlation analyses (Pearson/Spearman) were performed (significance *p* < 0.05). The main objectives of the study were to translate and adapt the POEM questionnaire into Romanian for use in children with AD and to assess the relationship between POEM and SCORAD scores. Secondary objectives were to explore the influence of clinical and perinatal factors (sex, prematurity, mode of delivery, in vitro fertilization, breastfeeding) and the presence of other atopic diseases on AD severity. Results: POEM and SCORAD scores showed a strong positive correlation (rho = 0.841, *p* < 0.001), indicating that patient-reported symptom burden closely paralleled clinician-assessed disease severity. The presence of another atopic condition was independently associated with higher POEM (B = 4.31, *p* < 0.001) and SCORAD scores (B = 15.34, *p* < 0.001). No other demographic or perinatal factor showed a consistent independent association with disease severity. No significant independent associations were found for sex, age, prematurity, in vitro fertilization, or breastfeeding. Conclusions: This first Romanian pilot study in pediatric AD demonstrates a strong correlation between POEM and SCORAD and supports the concurrent use of POEM as a practical patient-reported tool alongside SCORAD in clinical practice, while recognizing the limitations of a relatively small cohort and single-visit design.

## 1. Introduction

Atopic dermatitis (AD) represents an inflammatory, chronic skin disease, which evolves with episodes of exacerbation and is associated with itchy lesions and remission [1]. The epidemiology of this pathology varies depending on age. It affects 3% of adults and up to 20% of children; however, the incidence is also different in Europe (10–20%), while in the United States it is 17% [2,3]. On the other hand, the Western lifestyle seems to be another trigger, and is one of the factors that contributed to the increase in the incidence of atopic dermatitis after the 1960s [4]. The mechanism of the disease is complex: genetic factors, as well as dysfunction of the immune system Th2 (with production of IL4, IL5, IL13), decreased gamma interferon and increased IgE, alongside skin barrier impairment [5]. Other trigger factors include air pollution (dioxins), inappropriate diet, obesity, psychological stress, vitamin D deficiency, birth in winter/autumn, bathing in hard water, and perhaps even cesarean delivery; however, exposure to dogs seems to decrease the risk of atopic dermatitis [6,7]. As for the onset age, AD usually manifests in the first year of life (0–6 months) (60% of cases) [8]. AD affects quality of life. This is the reason why close follow-up should be performed for itching, sleep, and daily activity [9].

In order to follow-up these pediatric patients, two crucial tools are often used. First, with the aim of quantifying the impact of the disease on the lives of pediatric patients with atopic dermatitis, Patient- Oriented Eczema Measure (POEM) represents a useful option. On the other hand, clinical assessment should always be performed, such as by calculating the SCORAD score [10]. Several perinatal factors, including prematurity, mode of delivery, infant feeding practices, and assisted reproduction, have been investigated as potential modifiers of atopic disease risk, although findings remain inconsistent.

Preterm birth represents delivery before 37 weeks of gestational age. Also, assisted reproductive technologies, advanced maternal age and smoking may contribute to premature birth [11,12]. Currently, the data in the literature regarding the direct association between premature birth and the severity of atopic dermatitis are contradictory [13,14,15,16]. While the paradigm has been that babies’ intestines are sterile until birth, recent work found a microbial community already dwelling in the meconium of some babies born prematurely. It has also been shown that amniotic fluid of mothers with preterm labor contains a large and diverse spectrum of bacterial rDNA. Whether the microbes or microbial components swallowed in the amniotic fluid stimulate an inflammatory response driving preterm birth remains to be evaluated [17,18].

### Aim of the Study

The aim of the study is to translate and adapt the POEM (Patient-Oriented Eczema Measure) into Romanian for use in children with atopic dermatitis.

The main objective of our pilot study was to establish if an association exists between the POEM score and the severity of AD, quantified by SCORAD score.

The secondary objective was to explore whether selected demographic, perinatal, and clinical characteristics were associated with disease severity.

## 2. Materials and Methods

### 2.1. Study Design and Participants

The data for this observational analytical study were obtained from pediatric patients diagnosed with atopic dermatitis, who were recruited from Saint Constantin Hospital, Brasov, Romania, between September 2025 and February 2026. The study followed inclusion and exclusion criteria. Inclusion criteria were: patients under 18 years of age, diagnosed with atopic dermatitis of any form, after Hanifin and Rajka criteria. Major criteria were: pruritus, early age of onset, chronic dermatitis, typical morphology and distribution, personal or family history of atopy. Minor criteria included xerosis, pityriasis alba, nipple eczema, hand/foot-specific dermatitis, white dermographism, and susceptibility to cutaneous infections.

Exclusion criteria: Patients under 18 years of age, with any other form of eczema; other autoimmune skin diseases; infectious diseases; and pediatric patients whose caregivers did not respond to all questions were excluded from the study. In total, 90 pediatric patients aged 1 month–15 years, who presented with typical lesions for AD at the beginning of the study, were selected.

### 2.2. Clinical Evaluation

The impact of AD on pediatric patients was assessed using the POEM questionnaire. It quantifies the quality of life impairment of the child in the preceding week. Also, the group was divided in 2 smaller groups: younger than 5 years old (the caregiver answered for the child), and over 5 years old (the child answered with the caregiver). The POEM questionnaire has 7 items, covering the following 7 aspects of the impact of the eczema on the quality of life during the past week: itch, sleep disturbance, bleeding of eczema, weeping or oozing eczema, crack and flacking because of eczema, and dry or rough skin. The maximum POEM score is 28, with the following degrees of severity: 0–2 is clear or almost clear; 2–7 is mild eczema; 8–16 is moderate eczema; 17–24 is severe eczema; and 25–28 is very severe eczema. The POEM questionnaire was translated from English to Romanian, and then translated back into English by an authorized translator.

During the same visit, we completed the SCORAD score in order to quantify and classify the form of AD. For this score, we used the SCORAD calculator, which includes the area involved (body surface area); the intensity of the eczema (for this, a representative area is selected): redness, swelling, oozing/crusting, scratch marks, lichenification, and dryness; and subjective symptoms: a visual analog scale is used for itch and sleeplessness.

The two questionnaires were completed during the medical visit. The POEM questionnaire was paper and pencil-based, while the SCORAD score was directly calculated using scoring atopic dermatitis application by the dermatologist.

In order to be able to compare the preterm children with term children, the following data were also recorded: sex, age, preterm or term newborn, other associated atopic diseases, cesarean section compared with vaginally delivered children, in vitro fertilization, and newborn breastfed or formula.

### 2.3. Statistical Analysis

Descriptive statistics were used to characterize the sample. We first evaluated the distribution of data for POEM (non-normally, Shapiro–Wilk < 0.05) and SCORAD (normally, Shapiro–Wilk > 0.05). We then compared the mean scores of POEM and SCORAD between demographic and clinical characteristics using Mann–Whitney (and *t*-test for SCORAD) and presented the results as mean ± standard deviation. Afterwards, we conducted separate linear regressions for POEM and SCORAD to determine possible confounders and presented the results with unstandardized B, 95% confidence intervals and *p* value. Finally, given that in the current literature it is an ongoing debate regarding the impact of age on atopic dermatitis severity, we added a correlation analysis using Pearson (and Spearman for non-parametric data) and presented the data with the correlation coefficient and *p* value. All analyses were conducted using SPSS Statistics v26. Significance threshold was set at *p* < 0.05.

## 3. Results

### 3.1. Study Population

The study population comprised 90 pediatric children: 42 male (47.77%) and 48 females (52.23%), with a female predominance. As for the age, we divided the main group into two subgroups: under/and 5 year-old and over 5 year-old (65 patients—72.23%) and over 5-year-old (25 patients—27.77%).

Comparison between socio-demographic and clinical characteristics and mean POEM and SCORAD scores is shown in Table 1.

For children ≤ 5 years old, the value of the POEM score was 12.26 ± 0.65. For children > 5 years old, the value of the POEM score was 11.89 ± 1.02 (*p* = 0.793). There were no significant statistical differences regarding the mean POEM scores among the two age groups even though younger children developed slightly more severe forms.

As for the SCORAD, the data was similar between the two groups: 36.93 ± 1.88 (≤5 years old) and 36.69 ± 3.14 (>5 years old), with *p* = 0.923.

As for gender distribution, there was also no significant difference: the POEM score was 11.93 ± 0.80 (female) and 12.29 ± 0.76 (for male), with *p* = 0.755; on the other hand, the SCORAD mean was 35.65 ± 2.41 (for female) and 37.84 ± 2.17 (for male), with *p* = 0.501.

**Figure 1:**
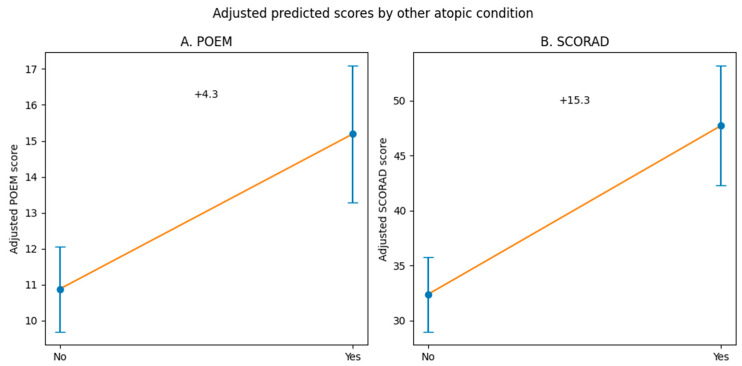
Adjusted predicted scores of (**A**) POEM and (**B**) SCORAD by the other atopic conditions variable.

However, what was highly statistically significant was the association of atopic dermatitis with other atopic diseases (see Figure 1). The most frequent was food allergy (13.5%), followed by rhinitis (8.88%), and then asthma (8.1%). As for the correlation with the scores, the data revealed that the POEM score was 15.62 ± 0.87 for association with allergic diseases versus 10.70 ± 0.60, for no other atopic diseases, with *p* < 0.01. The SCORAD scale showed similar results: mean ± SD was 48.28 ± 1.98 for association with allergic diseases versus 32.17 ± 1.83 for no other atopic diseases, with *p* < 0.01.

**Figure 2:**
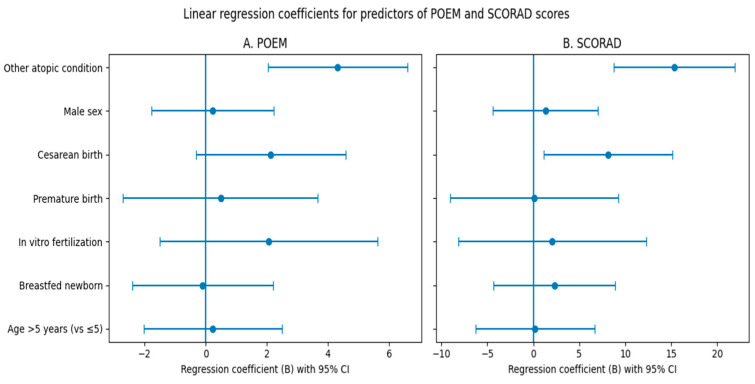
Linear regression coefficients for predictors of (**A**) POEM and (**B**) SCORAD scores with 95% CI.

The next socio-demographic factors were investigated in order to establish a correlation between these and the severity of atopic dermatitis. Although we could not find a relevant correlation, some differences were present (see Figure 2). The POEM score was 12.86 ± 0.63 for children born by cesarean section, versus 9.71 ± 0.94 for children born transvaginal, with *p* = 0.014. We found similar results for the SCORAD score, which was 39.19 ± 1.79 for children born by cesarean section versus 29.05 ± 3.15 for children born transvaginal, with *p* = 0.07.

We also investigated prematurity, as it may influence the severity of the disease: the POEM score was 14.52 ± 0.97 for preterm children versus 11.39 ± 0.63 for term children, with a *p* = 0.011. The SCORAD mean was 41.52 ± 2.86, versus 35.39 ± 1.89 for term children, with *p* = 0.108.

In vitro fertilization does not seem to influence the disease evolution and severity, but some differences were found: the POEM score was 15.36 + 1.23 for in vitro newborns, versus 11.67 ± 0.58 for the rest of them, with *p* = 0.016, while the SCORAD mean was 42.10 ± 4.34 for in vitro newborns, versus 36.09 ± 1.73 for the others, with *p* = 0.224.

Breastfeeding did not seem to influence the atopic disease: POEM score was 11.29 ± 0.82 for breastfed versus 12.85 ± 0.72 for milk formulas, with *p* = 0.072; SCORAD score was 36.08 ± 2.42 for breastfed, versus 37.47 ± 2.18 for the other, with *p* = 0.669.

### 3.2. Linear Regression for POEM Scores

Multivariable linear regression analysis showed that the presence of another atopic condition was independently associated with higher POEM scores (B = 4.31, 95% CI 2.03 to 6.60, *p* < 0.001).

No significant associations were observed for sex (B = 0.22, *p* = 0.823), cesarean section (B = 2.13, *p* = 0.086), prematurity (B = 0.49, *p* = 0.763), in vitro fertilization (B = 2.05, *p* = 0.256), and breastfeeding (B = −0.10, *p* = 0.934), or age > 5 years (B = 0.23, *p* = 0.837). (Table 2).

### 3.3. Linear Regression for SCORAD Index Scores

In the multivariable model, the presence of another atopic condition was strongly associated with higher SCORAD scores (B = 15.34, 95% CI 8.78 to 21.89, *p* < 0.001).

Additionally, cesarean section was independently associated with increased SCORAD scores (B = 8.12, 95% CI 1.13 to 15.11, *p* = 0.023).

No significant associations were found for sex (B = 1.33, *p* = 0.645), prematurity (B = 0.11, *p* = 0.981), in vitro fertilization (B = 2.05, *p* = 0.690), breastfeeding (B = 2.29, *p* = 0.493), or age > 5 years (B = 0.20, *p* = 0.951).

On the other hand, there was a statistically relevant correlation between SCORAD and the association of atopic dermatitis with other atopic diseases: B = 15.34, 95% CI 8.78 to 21.89, *p* < 0.001.

In the following sections, we present the evolution of some of the most representative patients with AD; POEM is correlated with SCORAD score (Table 3).

### 3.4. Correlation Between Total POEM and SCORAD Scores

A Spearman correlation analysis revealed a strong positive correlation between total POEM and SCORAD scores (rho = 0.841, 95% CI 0.768 to 0.893, *p* < 0.001), confirming that patient-reported symptom burden closely tracks clinician-assessed disease severity.

## 4. Discussion

To our knowledge, this is the first Romanian study evaluating the relationship between POEM and SCORAD scores in children with atopic dermatitis.

Overall, our findings demonstrated a strong association between patient-reported symptoms, assessed using the POEM score, and clinician-assessed disease severity, measured by SCORAD. Children with more severe forms of atopic dermatitis tended to have higher POEM scores, reflecting a greater symptom burden. These findings are consistent with previous studies conducted in pediatric patients with atopic dermatitis that used quality-of-life instruments rather than POEM, suggesting that increased disease severity is associated with a substantial impact on patients’ daily lives and well-being [19,20,21,22].

Although several demographic and perinatal characteristics were explored in the present study, the pilot design and limited sample size restrict the interpretation of these secondary analyses. Therefore, the findings should primarily be viewed as hypothesis-generating and require confirmation in larger prospective cohorts.

The connection between prematurity and atopic dermatitis (AD) is an intensively investigated topic with existing knowledge gaps. A meta-analysis was conducted in this area, including thirteen articles with a total of over 4 million participants, and in most of the studies (n = 8), prematurity was associated with a lower risk of atopic dermatitis [23]. There is a prospective birth cohort study that showed that preterm birth was associated with a significantly decreased risk of developing atopic dermatitis during the first year of life, as well as a decreased severity of atopic dermatitis at onset. Overall, the study confirmed previous risk factors for atopic dermatitis but identified slight differences between preterm- and term-born children. Prematurity was associated with older age at AD onset as well as lower AD severity [7].

Another study did not find a significant correlation between preterm and term pediatric patients with food allergies or AD. Also, it seems that the same results were found in neonates born by cesarean section or transvaginal [16].

The findings of this study should be interpreted in light of several limitations. The sample size was relatively small and reflected the pilot nature of the study, which limited the statistical power available for subgroup analyses and secondary exploratory comparisons. Consequently, the study was not specifically designed to robustly evaluate the influence of individual perinatal or demographic characteristics on disease severity. In addition, the cross-sectional single-visit design precluded assessment of disease evolution over time and prevented conclusions regarding temporal relationships. Recruitment from a single center may also limit the generalizability of the findings. Therefore, the observed associations involving secondary clinical and perinatal variables should be considered exploratory and hypothesis-generating, requiring confirmation in larger prospective multicenter studies.

## 5. Conclusions

This pilot observational study represents the first one published investigating the correlation between POEM and SCORAD scores in Romanian children with atopic dermatitis. The two scores were strongly correlated. Apart from the presence of concomitant atopic diseases, no consistent independent associations were observed between the evaluated demographic or perinatal characteristics and disease severity. These exploratory findings should be interpreted cautiously, given the pilot nature of the study. However, other atopic diseases, like asthma, were correlated with more severe forms of AD. Our findings should be interpreted in light of several limitations: a relatively small cohort, a lack of long-term follow-up, and the inability to include all pediatric atopic dermatitis patients because of incomplete data.

## Figures and Tables

**Table 1:** Comparison between socio-demographic and clinical characteristics and mean POEM and SCORAD scores.

Item	POEM Mean ± SD	*p*-Value	SCORAD Mean ± SD	*p*-Value
Age				
≤5 years	12.26 ± 0.65	0.793	36.93 ± 1.88	0.923
>5 years	11.89 ± 1.02		36.69 ± 3.14	
Sex				
Female	11.93 ± 0.80	0.755	35.65 ± 2.41	0.501
Male	12.29 ± 0.76		37.84 ± 2.17	
Other atopic disease				
Yes	15.62 ± 0.87	<0.001	48.28 ± 1.98	<0.001
No	10.70 ± 0.60		32.17 ± 1.83	
Cesarean section				
Yes	12.86 ± 0.63	0.014	39.19 ± 1.79	0.007
No	9.71 ± 0.94		29.05 ± 3.15	
Born prematurely				
Yes	14.52 ± 0.97	0.011	41.52 ± 2.86	0.108
No	11.39 ± 0.63		35.39 ± 1.89	
In vitro fertilization				
Yes	15.36 ± 1.23	0.016	42.10 ± 4.34	0.224
No	11.67 ± 0.58		36.09 ± 1.73	
Newborn breastfed				
Yes	11.29 ± 0.82	0.072	36.08 ± 2.42	0.669
No	12.85 ± 0.72		37.47 ± 2.18	

**Table 2:** Linear regression for POEM scores.

Item	B (Unstandardized) (95% CI)	*p*
Sex (ref: Female)	0.22 (−1.77 to 2.22)	0.823
Other atopic (Ref: No)	4.31 (2.03 to 6.60)	<0.001
Cesarean section (Ref: No)	2.13 (−0.31 to 4.57)	0.086
Born prematurely (Ref: No)	0.49 (−2.70 to 3.67)	0.763
In vitro fertilization (Ref: No)	2.05 (−1.51 to 5.62)	0.256
Newborn breastfed (Ref: No)	−0.10 (−2.40 to 2.21)	0.934
Age (Ref: ≤5 years)	0.23 (−2.02 to 2.49)	0.837

**Table 3:** Linear regression for SCORAD index scores.

Item	B (Unstandardized) (95% CI)	*p*-Value
Sex (ref: Female)	1.33 (−4.39 to 7.04)	0.645
Other atopic (Ref: No)	15.34 (8.78 to 21.89)	<0.001
Cesarean section (Ref: No)	8.12 (1.13 to 15.11)	0.023
Born prematurely (Ref: No)	0.11 (−9.03 to 9.25)	0.981
In vitro fertilization (Ref: No)	2.05 (−8.16 to 12.27)	0.690
Newborn breasted (Ref: No)	2.29 (−4.33 to 8.90)	0.493
Age (Ref: ≤5 years)	0.20 (−6.25 to 6.65)	0.951

## Data Availability

The de-identified dataset supporting the conclusions of this article is available from the first author upon reasonable request.

## Abbreviations

The following abbreviations are used in this manuscript:

AD	atopic dermatitis
CB	cesarean birth
VD	vaginal delivery
FA	food allergy
Staf. aureus	Staphylococcus aureus
AMP	antimicrobial peptides

## References

[1] Kim J.P., Chao L.X., Simpson E.L., Silverberg J.I. (2016). Persistence of atopic dermatitis (AD): A systematic review and meta-analysis. J. Am. Acad. Dermatol..

[2] Larsen F.S., Hanifin J.M. (2002). Epidemiology of atopic dermatitis. Immunol. Allergy Clin. N. Am..

[3] Laughter D., Istvan J.A., Tofte S.J., Hanifin H.M. (2000). The prevalence of atopic dermatitis in Oregon schoolchildren. J. Am. Acad. Dermatol..

[4] Monti F., Agostini F., Gobbi F., Neri E., Schianchi S., Arcangeli F. (2011). Quality of life measures in Italian children with atopic dermatitis and their families. Ital. J. Pediatr..

[5] Sur M., Boca A.N., Ilies R.F., Floca E., Tataru A., Sur L. (2020). Correlation between quality of life and disease severity of pediatric patients with atopic dermatitis. Exp. Ther. Med..

[6] Weidinger S., Novak N. (2016). Atopic dermatitis. Lancet.

[7] Gerner T., Rinnov M.R., Halling A.-S., Ravn N.H., Knudgaard M.H., Ewertsen C., Trautner S., Jakasa I., Kezic S., Skov L. (2022). Differences in Occurrence, Risk Factors and Severity of Early-onset Atopic Dermatitis among Preterm and Term Children. Acta Derm. Venereol..

[8] Spergel J.M., Paller A.S. (2003). Atopic dermatitis and the atopic march. J. Allergy Clin. Immunol..

[9] Kage P., Simon J., Treudler R. (2020). Atopic dermatitis and psychosocial comorbidities. J. Der Dtsch. Dermatol. Ges..

[10] Stefanou G., Gregoriou S., Bakakis M., Mastraftsi S., Stratigos A., Kontodimas S., Sfaelos K., Kourlaba G. (2023). Translation and validation of patient-oriented eczema measure in the Greek language. Dermatol. Rep..

[11] Cha J.H., Hwang J.K., Na J.Y., Ryu S., Oh J.W., Choi Y.J. (2024). Association between preterm birth and asthma and atopic dermatitis in preschool children: A nationwide population-based study. Eur. J. Pediatr..

[12] Goldenberg R.L., Culhane J.F., Iams J.D., Romero R. (2008). Epidemiology and causes of preterm birth. Lancet.

[13] Barbarot S., Gras-Leguen C., Colas H., Garrot E., Darmaun D., Larroque B., Roze J., Ancel P. (2013). Lower risk of atopic dermatitis among infants born extremely preterm compared with higher gestational age. Br. J. Dermatol..

[14] Trønnes H., Wilcox A.J., Lie R.T., Markestad T., Moster D. (2013). The association of preterm birth with severe asthma and atopic dermatitis: A national cohort study. Pediatr. Allergy Immunol..

[15] Egeberg A., Andersen Y.M., Gislason G., Skov L., Thyssen J.P. (2016). Neonatal risk factors of atopic dermatitis in Denmark—Results from a nationwide register-based study. Pediatr. Allergy Immunol..

[16] Kvenshagen B., Jacobsen M., Halvorsen R. (2009). Atopic dermatitis in premature and term children. Arch. Dis. Child..

[17] Mshvildadze M., Neu J., Shuster J., Theriaque D., Li N., Mai V. (2010). Intestinal microbial ecology in premature infants assessed with non-culture-based techniques. J. Pediatr..

[18] DiGiulio D.B., Romero R., Amogan H.P., Kusanovic J.P., Bik E.M., Gotsch F., Kim C.J., Erez O., Edwin S., Relman D.A. (2008). Microbial prevalence, diversity and abundance in amniotic fluid during preterm labor: A molecular and culture-based investigation. PLoS ONE.

[19] Ricci G., Bendandi B., Bellini F., Patrizi A., Masi M. (2007). Atopic dermatitis: Quality of life of young Italian children and their families and correlation with severity score. Pediatr. Allergy Immunol..

[20] Weidinger S., Simpson E.L., Silverberg J.I., Barbarot S., Eckert L., Mina-Osorio P., Rossi A.B., Brignoli L., Mnif T., Guillemin I. (2024). Burden of atopic dermatitis in paediatric patients: An international cross-sectional study. Br. J. Dermatol..

[21] Kisieliene I., Mainelis A., Rudzeviciene O., Bylaite-Bucinskiene M., Wollenberg A. (2024). The Burden of Pediatric Atopic Dermatitis: Quality of Life of Patients and Their Families. J. Clin. Med..

[22] Ohya Y., Saeki H., Nawata H., Arima K., Inukai M., Rossi A.B., Le-Bagousse-Bego G. (2023). The disease burden of pediatric patients with atopic dermatitis in Japan. Pediatr. Dermatol..

[23] Kowalik A., Cichocka-Jarosz E., Kwinta P. (2023). Atopic dermatitis and gestational age—Is there an association between them? A review of the literature and an analysis of pathology. Adv. Dermatol. Allergol..

